# How individuals formulate their beliefs about chronic musculoskeletal pain: introducing the dual implicit-explicit processing (DIP) model of pain belief formation — a qualitative exploration

**DOI:** 10.1186/s12891-026-09835-5

**Published:** 2026-04-29

**Authors:** Michael Dunn, Alison B Rushton, Nicola R Heneghan, Andrew Soundy

**Affiliations:** 1 Centre of Precision Rehabilitation for Spinal Pain, School of Sport and Exercise and Rehabilitation Sciences, University of Birmingham, Edgbaston, Birmingham, B15 2TT UK; 2 School of Health and Medical Sciences, City St. George’s University of London, Cranmer Terrace, London, SW17 0RE UK; 3 School of Physical Therapy, Faculty of Health Sciences, Western University, Richmond Street, London, ON N6A 3K7 Canada

**Keywords:** Interpretative phenomenological analysis, Chronic musculoskeletal pain, Qualitative, Beliefs

## Abstract

**Background:**

Chronic musculoskeletal pain (CMP) is complex with many biopsychosocial factors that contribute to its development. Existing research has established many beliefs that individuals’ hold about their CMP, but the sources of information and mechanisms used to construct beliefs are not well understood. The aim of this study was to observe and describe the mechanisms and sources of information individuals use to formulate their beliefs about CMP.

**Methods:**

A preliminary exploration using interpretative phenomenological analysis methods is reported according to the Consolidated Criteria for Reporting Qualitative Research. Adults with CMP were recruited from the general public. Four stages of data analysis based on Smith and Osborn were undertaken to identify themes on the sources of information and mechanisms used to construct beliefs about CMP.

**Results:**

Individuals’ explicit (conscious) beliefs are grounded in their own research, knowledge, and experience, conversations with friends, family, or others in pain, and conversations with healthcare professionals informed by radiographic investigations. This information is synthesised into stories that make sense to the individual in understanding and explaining their CMP; narratives that are felt not to make sense are rejected as beliefs. When asked to justify beliefs, individuals checked against their own experiences or existing knowledge; if the narrative correlated with experiences or existing knowledge (e.g., wearing heel must contribute to CMP because it hurts while wearing them) then the narrative was justified as an accepted belief. Likewise, if narratives conflicted with own experiences or existing knowledge then it was justified as a rejected belief. Cognitive errors were evident with contradictory beliefs existing simultaneously, and narratives rejected as beliefs despite correlating with experiences or knowledge. These mechanisms explain how individuals formulate explicit, conscious beliefs, however, existing literature suggests that the majority of our beliefs are implicit, formulated automatically and unconsciously, driving motivations, attitudes and behaviours nonetheless. Therefore, we interpret our findings within this context, and propose the Dual Implicit-Explicit Processing (DIP) Model of Pain Belief Formation.

**Conclusion:**

The DIP model may be used by clinicians to target both implicit and explicit mechanisms to help individuals modify their beliefs in line with contemporary evidence, adopt helpful behaviours, and better manage CMP.

**Supplementary Information:**

The online version contains supplementary material available at 10.1186/s12891-026-09835-5.

## Background

### Introduction

Chronic musculoskeletal pain (CMP) is a leading cause of disability worldwide, affecting approximately 43% of the United Kingdom [1], and poses a substantial burden on individuals, healthcare systems, and economies across the world [2]. Despite advances in clinical guidelines and evidence-based interventions, management outcomes remain poor for many people who continue to experience unsatisfactory levels of pain and disability [3]. Research highlights that individuals’ beliefs about their pain – such as its causes, consequences, and effectiveness of different interventions – play a central role in how they experience, cope and manage CMP [4, 5].As such, beliefs about CMP can significantly influence health behaviours, treatment choices, and recovery trajectories [6, 7].

Research suggests that it is not the pain itself, but the meaning attributed to the pain that drives behaviours and subsequently predicts healthcare outcomes [8, 9]. For example, biomedical beliefs that pain is a sign of ongoing damage can lead to fear-avoidance, lower physical activity, and long-term disability, while conversely, beliefs aligned with a biopsychosocial understanding of pain are associated with better coping, higher self-efficacy, and improved outcomes [10–14]. Yet, there remains a widespread mismatch between contemporary scientific understandings of pain [15] and the beliefs held by many individuals living with CMP [5, 16]. Identifying the mechanisms by which individuals form their beliefs – and the sources they rely on to construct those beliefs – is therefore critical for designing effective interventions that support more accurate, empowering and evidence-aligned understandings of CMP.

Studies have highlighted that individuals draw upon diverse sources of information to make sense of their pain, including personal experience, family and peer narratives, social and cultural context, interactions with healthcare professionals, and media representations [17–20]. The construction of beliefs from these sources is a learning process. Beliefs are not innate but are learnt, and contemporary theories of learning suggest that learners actively construct knowledge by building upon their existing understandings via both internal (cognitive) and external (social – peers, family, and more knowledgeable others) methods [21]. Likewise, in the context of health, the Common-Sense Model of Self-Regulation suggests that individuals actively construct illness representations based on both internal (symptoms, experiences) and external (social and informational) cues [22, 23]. Deconstructing learnt beliefs and illness narratives is difficult with catastrophised beliefs about pain often remarkably persistent. For example, even after pain neuroscience education, Joern et al. [24] found that patients reverted to previously held fearful beliefs when pain did not fully resolve, or during a flare up. It may be that biomedical explanations orientated around structural vulnerability are more simple than pain science to understand, and therefore easier to reconcile with lived experience; easier to believe.

There appears to be something incredibly alluring and convincing about simple structural based explanations informed by radiographic imaging. For example, non-Westernised civilisations who traditionally hold more appropriate beliefs about CMP, such as pain being a natural part of life, shift to biomedical beliefs (structural vulnerability) in response to imaging and biomedical explanations [17]. Whilst pain being a natural part of life is accurate, this belief is abstract and not easy to explain. Structural vulnerability on the other hand *is* easy to explain and correlates with our experience of structural vulnerability of any number of objects or structures in the real world. Perhaps this is why it is easy to shift to biomedical beliefs, and hard to shift to pain neuroscience beliefs. Understanding this process in more depth is critical to inform best practice for changing beliefs about CMP.

Understanding how beliefs about CMP are formed—and what sources of information individuals rely on—is essential for bridging the gap between pain science and patient experience. While many studies have detailed patient beliefs about CMP, existing research has primarily focussed on describing what beliefs exist, the sources of information that inform beliefs, or development of beliefs in specific contexts [4, 5, 8, 16–18, 24, 25].To the best of our knowledge, no study to date has explicitly sought to describe the cognitive mechanisms that individuals use to develop and justify their beliefs. By uncovering this process, we can better support individuals to adopt or shift beliefs that align with contemporary pain science, and maximise better coping, higher self-efficacy, and improved outcomes for CMP.

## Methods

This paper presents part of the findings of a broader study exploring the beliefs of individuals living with CMP. The published study protocol [26] outlines two main aims; to understand what individuals’ beliefs are about the biopsychosocial factors that contribute to their CMP, and to understand how individuals formulate their beliefs. Data analysis revealed conceptually distinct findings between these two research aims. Therefore, the decision was made to present the findings across two research papers to preserve the nuance, depth and analytic complexity of the findings for each aim.

### Aims and objectives

To understand what sources and mechanisms individuals use to construct their beliefs about their CMP.

### Design and theoretical framework

This qualitative study was designed and reported using the Consolidated Criteria for Reporting Qualitative Research (COREQ) [27] (Additional File 1 – Completed COREQ) with the study protocol published a priori [26].

A preliminary exploratory study is a small-scale, initial investigation conducted when little is known about a topic, with a key aim being to clarify the nature of the phenomenon to help shape future research, conducted on a small scale with flexible designs [28]. This is particularly appropriate for gaining familiarity with a poorly understood problem such as how beliefs are constructed and formulated on the causes of CMP.

The research methods for this preliminary exploration are underpinned by Interpretive Phenomenological Analysis (IPA) and data was collected using semi-structured interviews. IPA is an appropriate methodology for studying the formulation of beliefs about CMP for several reasons. Firstly, IPA is grounded in phenomenology which emphasises how individuals make sense of their personal, lived experiences [29]; essential for understanding formulation of beliefs. IPA also takes an idiographic approach with a focus on in-depth experiences of a small number of participants which allows for detailed, nuanced understandings [29] which is especially important in CMP where beliefs about contributors may be deeply personal, and therefore mechanisms for formulating beliefs may be varied. Furthermore, the double hermeneutic process within IPA, whereby the researcher observes and interprets the participants sense-making of their own experiences [30], is crucial here because this allows the researcher to observe and detail mechanisms and biases within the belief formulation process which may not be apparent to the participant.

This research is situated within a constructivist research paradigm, a branch of interpretivism which places added emphasis on how individuals construct meaning through experience, interaction and dialogue within their own social and cultural contexts, with knowledge co-constructed through the interpretative lens of the researcher [31, 32].

### Sampling & recruitment

A purposive sample was used with the main criteria being presence of CMP without a clear cause. A sample size of 6–12 was planned in keeping with IPA methods to maintain depth of analysis [29]. Smaller sample sizes are common in qualitative research which values depth over breadth, and specifically, a sample size of 4–10 is recommended for IPA research [33–35]. Larger sample sizes are appropriate within quantitative research aiming for statistical generalisability; this is not the aim for qualitative research [36] which seeks to achieve naturalistic generalisability where the results have meaning if the “ring true” for the intended population [37].

Due to the preliminary exploration nature of the study, a maximum variation sample was intended to gain understandings of a broad range of beliefs of people from a rich variety of backgrounds which then may inform areas of focus for future research. This was achieved in terms of socioeconomics, but not ethnic or younger adults. Members of the public were made aware of the study through advertisement. This included advertising to a patient and public mailing list at the University of Birmingham, and was expanded to include circulation on social media (Twitter/X) including specialist interest groups (e.g., Pain UK on Facebook) to specifically target ethnic and younger adults, but this was not effective. These recruitment difficulties are dissected within the discussion, with recommendations made to overcome these barriers within future research. Potential participants who contacted the lead researcher (MD) via email were provided with a study Participant Information Sheet and were screened for eligibility with a telephone conversation.

### Semi-structured interviews

One semi-structured interview was conducted with each participant by the lead researcher (MD) who is male and was a practicing musculoskeletal physiotherapist with 10 + years’ experience in the NHS and a Masters of Research student at the time of interview. Participants were aware of these details about the interviewer and aware of the rationale for conducting the research. Interviews were all conducted within 3 weeks of obtaining informed consent and were conducted remotely using a secure online video platform (Zoom). The interview schedule was informed by the biopsychosocial model of health, an extensive umbrella review of the factors associated with development of CMP [38], the expertise of the authors (MD, AR, AS & NH) and input from patients and public through a meeting with a Patient and Public Involvement (PPI) group with CMP. The original interview schedule was tested with two pilot interviews which identified some issues with flow and ease of understanding of questions. This was therefore updated since publication in the protocol [26] prior to use with participants (Additional File 2 – Interview Schedule) without further amendment thereafter. Interviews were audio recorded and transcribed verbatim by the lead researcher. Field notes were taken. No prior relationship existed between participants and the lead researcher.

### Data analysis

**Fig. 1:**
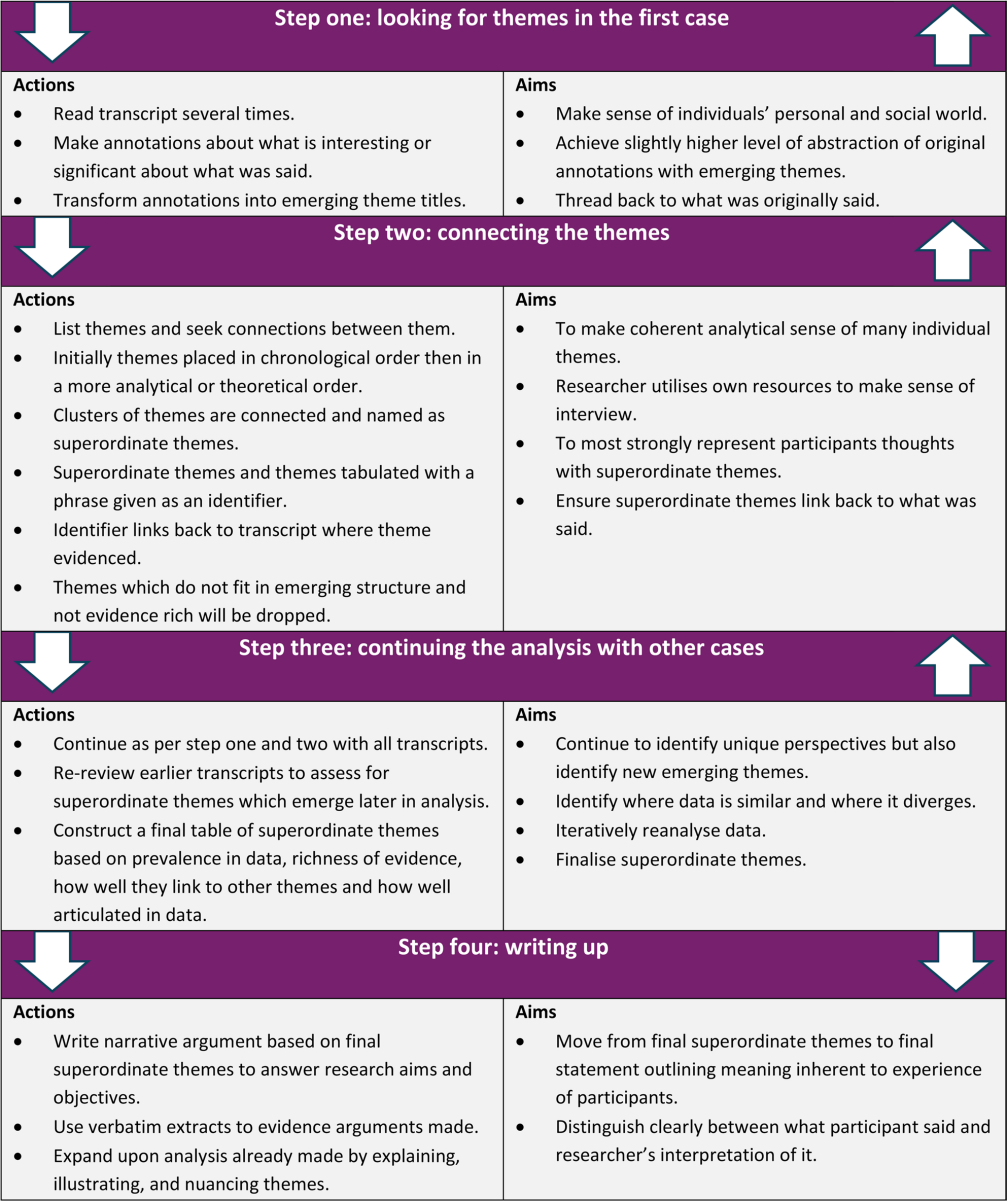
Interpretative phenomenological analysis methods based on Smith and Osborn, 2008 [26]

Four steps of analysis were based on Smith and Osborn [29] as published in the study protocol [26] (Fig. 1). ‘Data saturation’ is a concept commonly used in qualitative methods however it was not employed within this study as reaching an endpoint where no new knowledge can be ascertained, despite being a qualitative method, is more coherent with quantitative research philosophies and therefore incongruent with the values and assumptions of the constructivist paradigm within which this research is situated [39]. Analysis was considered to be complete upon achieving understanding and coherence whilst preserving nuance [30]. MD read each transcript several times and applied coding to identify preliminary themes. MD grouped themes under superordinate themes illustrated with direct quotations. Themes were then critically discussed and refined iteratively amongst all co-authors (MD, AS, AR & NH). Themes were inductively derived from the data. Themes and codes were managed using Microsoft Excel. Supporting quotations used for analysis are provided throughout the results with further quotes available in Additional file 3, and coding trees available in additional file 4.

### Strategies to ensure trustworthiness

A reflexive account of the dynamic research process throughout this study has been provided within this article [30], including acknowledgement of any limitations; the hermeneutic circle has been considered throughout interpretation for analysis [30]; ‘member checking’ has been performed [40]; and lastly, validation checks of interpretations including scrutinization of any preconceptions or biases, were considered through discussion and feedback between all authors [40].

### Ethical considerations

Informed consent was obtained from all participants [26]. Ethical approval was acquired from the Research Ethics Office at the University of Birmingham, United Kingdom (reference ERN_21–0813). Anonymity has been maintained; the names of participants used in this article are pseudonyms.

### Patient and public involvement (PPI)

PPI has been integral to the development of this research study. The lead researcher conducted an interactive discussion with members of the public with CMP from the Centre for Precision Rehabilitation for Spinal Pain PPI Group at the University of Birmingham as detailed within the published protocol [26].

## Results

### Participants

Ten potentially eligible participants were assessed for eligibility. Eight were eligible, *n* = 2 did not respond to further email communication following the screening phone call (no reasons provided) and *n* = 6 completed informed consent and participation. All interviews lasted 50–70 min and were conducted with interviewees at their own home with no one else present. See Table 1 for characteristics of participants, and Table 2 for a description of each participant’s CMP, vocation, and hobbies. CMP presentations across participants are similar with all six participants having multi-site CMP, including five participants with chronic lower back pain and four with chronic knee pain.

**Table 1:** Characteristics of participants (obtained from participants)

		*n*
Age, years	40–49	1
	50–59	3
	70–79	1
	80–89	1
Sex	Male	2
	Female	4
Current work status	Retired	1
	Unable to work due to CMP	1
	Employed full time	4
Highest educational award	College/sixth form	2
(or equivalent)	University undergraduate degree/qualification	2
	Doctoral	2
Current avg. household	£0-20k	1
income	£20-40k	2
	£60-80k	2
	£200k+	1
Ethnicity	White British	5
	White Jewish	1

**Table 2:** Details of participants CMP, vocation, hobbies and activities

“Charlotte”	Chronic lower back and bilateral knee pain, starting 20 years ago. Retired secondary school science teacher. Used to enjoy long walks, now does shorter walks, swims and does embroidery
“Tony”	Chronic lower back, neck, bilateral ankle, wrist, and knee pain, fibromyalgia, starting 20 years ago. Ex British Armed Forces, then IT engineer, now medically retired due to CMP; attends Help for Heroes meetings and veterans club, but spends time at home mostly due to pain
“Catherine”	Chronic lower back and leg pain, bilateral fingers, hand and foot pain, starting 30 years ago. Works as a doctor (previously GP, now occupational medicine). Goes to the gym, enjoys arts and crafts
“Bethany”	Chronic lower back, neck, hip, bilateral knee, bilateral hand pain, fibromyalgia, starting 22 years ago. Previously worked as a community carer, now a school trauma counsellor. Previously very active with “mum stuff” (walking dogs, park with kids, playing, walking, household tasks) but now can’t do this, swims occasionally if able, but spends time at home mostly due to pain
“Hannah”	Chronic neck, bilateral shoulder and arm pain, fibromyalgia, starting 7 years ago. Previously worked as a teacher (describes as “burnt out teacher”), now does desk-based work in nature conservation. Used to cycle, run and swim, now does yoga, walks dog, does arts and crafts, continues to swim
“Edward”	Chronic lower back, bilateral knee, wrist, finger and toe pain, starting 20 years ago. Previously a teacher and teacher trainer, now semi-retired teaching consultant. Does long walks, volunteers in teaching associations and public research involvement

### Implicit and explicit beliefs

A belief may be considered as cognitive representations of information, opinions, or expectations about the world that are presumed to be true [41]. However, belief systems are far more complex with far wider implications than this superficial understanding allows. Belief systems form our own individually constructed frameworks for interpreting the world and our place within it, and serve as the foundations for all of our understandings, motivations and our behaviours [42]. Every action we take is grounded in an elaborate web of beliefs, for example, the simple act of opening a door requires beliefs in what a door is, what lies behind the door, our ability to use a door, and our beliefs in the possible outcomes of using or not using this particular door, at this particular time, in this particular context [43]. Due to the cognitive burden of consciously computing this complex web of detail, it is understood that the majority of beliefs, understandings, and reasons for behaviours remain unconscious or outside of our immediate awareness, with individuals having little insight into the mechanisms of this process [44–46]. In acknowledgement of this, our results focus on the mechanisms observed for explaining and justifying explicit, consciously held beliefs about CMP. As part of the double hermeneutic, we then interpret our findings within the context of what is known about implicit, unconscious mechanisms for formulating beliefs outlined in other well established psychology studies. 

### How explicit beliefs about CMP are formulated

#### Sources of information: the self, others, professionals and imaging

Individuals outlined numerous beliefs about their CMP and why they have it. Predominantly, individuals believed that degeneration or injury to MSK structures were the main explanations for their having CMP, and that physical activities and postures were the cause of this. They believed that negative psychological factors (e.g., emotional distress, depression, etc.) did not contribute to CMP, but did believe that positive psychological factors (e.g., reassurance) contributed to reduced severity of CMP. Participants attributed the underlying information for these beliefs to a range of sources. Almost all participants directly attributed their beliefs to their own knowledge and experience, including research on the internet. Explaining the basis for beliefs, participants stated:

“Just what I live through every day. And I did the research on degenerative discs when I first got diagnosed with it… and I listen to my body quite a lot. So, it’s listening to my body and just being aware of myself.” (Bethany)

“It comes from my experience, from my background. From my you know, my knowledge, you look up things. Before Google, you would look it up in the book and find out.” (Charlotte)

“Experiences, yeah… learning what’s good and what’s bad for you as a person, because everybody is different… So you know the certain sports that you can’t do. You test them out, that’s what they do all these things for, so you test it out, ‘how is that?’, that’s not for me, you know”. (Tony)

“I just do a lot of research, you know. Google is a fantastic thing nowadays to find out research that’s been done on a certain thing, and you know you can get a better understanding now than before. Say my condition, you know. You look at something like you’re trying to diagnose a condition that you’ve got… But then you read more into it, study more, and you think, okay, it looks more like this.” (Tony)

Participants also attributed the sources of their beliefs to discussions with other people:

“Before Google, you would look it up in the book and find out, or ask people. I have a brother who was a senior consultant, GP nephews and nieces.” (Charlotte)

“the only reason why I went down a diagnosis route for that is because a friend, cause she had seen me at Invictus training camp, and she’s saying to her husband ‘I think that Tony has got fibro’ etc., etc., you know. So then we had a good talk about it.” (Tony)

“It helps talking to other people in the same boat with chronic illness, like on Facebook, living with chronic pain.” (Bethany)

Information given by healthcare professionals informed by imaging appeared to be an extremely salient source of beliefs with every participant identifying a biomedical diagnosis for their CMP identified through a scan:

“I had a few hospital stays where they’ve had to put morphine in me to get me into the ambulance, and do a few more scans. Now it’s gone up to my T7, T8, and in that time I got bursitis in my left hip. That’s when they found I got arthritis in that hip, and my knees, and my hands.” (Bethany)

“In 2017 I had a big flare up. So I went to the doctors and they sent me for an MRI, and that’s when they discovered that I’ve got degenerative disc disease in my L4 and L5.” (Catherine)

“And now I got my MRI, was yeah, the L 2, 3, 4, 5, and S1 bulging.” (Tony)

Having been shown MRI images of her neck: “They’re pressing on something. And so yeah, I couldn’t say. But having seen them, and seeing that they’re bulging on to something that is triggering pain. Yeah, it must contribute to how I feel.” (Hannah)

Participants appeared to synthesise these building blocks of information into their own story that makes sense to them in explaining their CMP.

“I do have some knowledge of biology. And I do look things up on the Internet sometimes. Suppose the Internet wasn’t around at the time that these things became my problems. But I suppose I could have read about it, probably talked about it, at least with the GP in the case of the knees, who may have said, and I’ve had time to say that the cause is, such and such. A lot is guesswork I think.” (Edward)

#### Stories that make sense

Participants explained the causes of their CMP in the form of a narrative which made sense to them in explaining their pain. They used their own understandings and their own experiences of the world in order to justify their story about their CMP. Individuals’ experiences of the world vary widely, and therefore the experiences and knowledge used also varied. All participants explained their CMP stories through concepts such as biomechanics, pressure/compression, and past experiences of injuries and healing:

“the vertebrae were not even… And so, you ended up with presumably cartilage on cartilage, but that has presumably worn away, and you’ve got bone rubbing on bone. That would be my interpretation of it.” (Charlotte)

“They’re pressing on something. And so yeah, I couldn’t say. But having seen them and seeing that they’re bulging on to something that is triggering pain. Yeah, it must contribute to how I feel”. (Hannah)

“I think it gets worse over time. It’s like anything; if you’ve got a cut and you don’t put a plaster on, it’s gonna bleed, you know. And if you leave it over time it can heal, but sometimes it can leave a callous there. So you know, it’s not fully healed (Tony)

But participants also described their CMP through their varied understandings of biology, musculoskeletal structures, injuries or degenerative changes:

“So, to my knowledge degenerative discs is the discs in between obviously degenerate, so they’re crumbling, and I know that one of my discs are quite low, so it’s near bone on bone. So if I move funny, the pain goes through my whole body, and then I obviously get bulging discs. So I know it’s down to the nerves in my body that have been compressed or irritated. Well, that’s my understanding, anyway.” (Bethany)

“Well, they called it a slipped disc. I don’t know if they still use the term slipped disc. As I understand it, that’s the cushioning between two vertebrae that pops out of alignment and needs to be restored back into position.” (Edward)

“And that’s when I found out, actually, I’d got some slipped discs. So, the pain I had for that was just horrible… Oh I’ve got spondylosis as well, I forgot about that.” (Hannah)

A key component of these stories are that they make sense to the *individual* who tells it. As such, the level of complex critical thought applied to individuals’ understandings of their CMP varied with one participant exhibiting a higher level of comprehension by considering that musculoskeletal structures are living organisms that can repair:

“I believe it’s wear and tear on the joints, particular parts of the joints, the cushions of the joints, or something, I don’t know. But as these are living things, they presumably have the power to keep themselves repaired as much as possible… So I believe that usage does continue to help the repair process, and non-usage tends to encourage it not to repair.” (Edward)

This insight also had the positive benefit of making sense of why movement and usage of MSK structures is helpful for managing CMP. Other participants who made sense of their CMP through biomechanics and tissue stress also believed that movement and activity contributed to CMP:

“So there’s a lot of heavy lifting, a lot of jumping. So all that definitely did go to my back pain.” (Tony)

“The bursitis in my hip is when my bursa sac gets inflamed. And that can be because of the arthritis or because I’ve moved funny or overdone stuff.” (Bethany)

“I mean looking back on it now, the slipped discs or the bulging discs just didn’t happen overnight. I must have been making them worse over a series of years, so I definitely think, not being a natural runner anyway, I took up running quite late, I was in my late thirties, I think… Yeah, I definitely think cycling, will have, I don’t think it caused it, but it will have definitely contributed” (Hannah)

#### Correlation with experience or knowledge

This step did not arise organically during participants’ explanations of their beliefs, but only when asked why they believe their CMP stories to be true. Participants demonstrated this further mechanism in order to justify holding their belief; they assessed the information against their own existing knowledge or experience. If the information was easy to correlate (i.e., did not cause any cognitive conflict or imbalance) then it was generally accepted as a belief. A common method of justifying a belief was whether pain was worse or better during the activity or experience:

“It’s to do with wearing heels, I think, because that’s when I noticed it.” (Catherine)

Participants also correlated their CMP with their experiences of being active earlier in their lives. They would often make connections with their CMP to the activity, and then justify how that activity may have caused a biological structural change, such as injury or degeneration, via their understandings of biomechanics and pressure:

“it’s just a bit of a mystery as to why my discs are in the mess that they are, because I’ve had no specific injury, but I have theories as to where it might have come from. I did have a skiing accident as a teenager where I attempted some moguls that I wasn’t very good at, and ended up coming back in with my neck and a collar. I was about 17, but as far as I know, there was no damage done, but who knows?” (Hannah)

“So you don’t get down from a tank, you jump down from a tank. Yeah, you would never get down and it’s all this sort of thing because you get shouted at because you’re going too slow. Because, yeah, you know, you’ve got to be quick, quick, quick, quick. It’s all called shooting scoops… So yeah, all that. Yeah, has gone to the back pain. And now I got my MRI, was yeah, the L 2, 3, 4, 5, and S1 bulging, and this and this and this. So, yeah, from that, all that compression I think you’re doing.” (Tony)

If the information being discussed was difficult to correlate with participants own existing knowledge or experiences (i.e., the information caused cognitive conflict or imbalance) then it was generally rejected as a belief. One participant described being very stressed through periods of her life but did not think this was related to her CMP:

“So, the time pain has been worse hasn’t correlated with times of increased stress, I would say.” (Catherine)

Another participant described many negative psychological factors related to her CMP, but was not able to make sense of how this would make her CMP worse, and therefore rejected the information as a belief:

“I just think it’s the compression. I think it is down to the nerves, and the biology of it all. I don’t think it’s down, I don’t think it’s a psychological issue. I honestly do think it’s down to, when I like tense up, so I can feel, just now I feel the pull.” (Bethany)

#### Cognitive error

There appeared to be many instances where individuals exhibited cognitive errors in justifying their beliefs. Cognitive errors (also known as biases or distortions) are faulty mechanisms of thinking that can skew how individuals interpret information or experiences leading to errors such as holding contradictory beliefs simultaneously, which are common in individuals interpretations of their chronic conditions [47].

Tony and Bethany both stated they do not believe their negative psychological experiences influenced their CMP but separately describe instances where psychological experiences did influence their CMP. Tony, talking about the impact of his negative psychological experiences on his CMP stated.

“I don’t think they knock on as much. They probably do affect the mental side more than the physical. Definitely, yes, but yeah, not the other way around.” (Tony)

But later, reflecting on the positive psychological experience of participating in Invictus training, stated:

“When you’re focussed on something and you’re feeling, you’ve got them endorphins going around, you know, pain goes away… you’re concentrating on the good things, the things that’s making you happy; the pain goes away.” (Tony)

Catherine, a GP, who has dealt with bouts of high stress appeared to notice instances where her understandings contradicted other beliefs or previous statements based on her knowledge from being a doctor, but re-committed to the belief nonetheless:

In response to being asked why stress doesn’t affect her CMP: “I’m not sure, because that’s not what I see in everyday life, you know, in my professional life I see that stress makes pain worse [for patients].” (Catherine)

“So, I think it’s mainly degenerative, but obviously not in my thirties, I’m not sure, I’m not sure why my, well, no, I still think it’s just progressive and degenerative in nature.” (Catherine)

Most participants held contradictory beliefs that physical activities contribute to CMP but simultaneously believe that exercise is helpful for managing CMP:

Catherine, believes exercise is essential for managing her CMP: “If I don’t do exercise, my back pain is worse.”, but also believes that walking may have contributed to its development: “I’ve always wondered at the back of mind, if I’m making it worse by, because I do a lot of walking.” (Catherine)

Charlotte notes that remaining active helps manage her CMP: “It’s much better when I’m able to keep active. If I’m not active, I would seize up.”, but also stopped walking out of concern for making it worse: “We used to go walking quite a lot until the last, probably 15 years. My husband does. He still does that. But I gave that up really.” (Charlotte)

Tony is confident that activity helps to manage CMP: “if you’re active regardless of what that activity is, anything is good for the more in the mind and the body. I think, well, I’ve come to learn, because of doing Invictus and everything else, doing the training on the trials and all that, it does make you better, because it’s a double whammy. It’s a mental and a physical euphoria to do something like sports”, but remains inactive “I’m not mobile at the minute. If I need to go anywhere, I got the wheelchair if I need to.” (Tony).

This dissonance may exist due to the disconnect between personal experience and biomedical beliefs. In the statements above, participants outline personal experiences of exercise and activity improving their CMP, but this is not compatible with their beliefs about biomechanics, tissue stress and compression causing structural injury or degeneration. So, the two incompatible beliefs exist simultaneously and separately.

Cognitive errors, as detailed above, are a normal and known type of thinking mistake that all people exhibit that leads to flawed beliefs, judgments and decisions. In the examples provided above, these beliefs are observed to be flawed because they exist in direct contradiction to other beliefs. Cognitive errors like these can occur due to a number of cognitive biases such as confirmation bias, attention bias, or recall bias, amongst many others [47]. For example, Tony’s cognitive error about psychological experiences could be caused by cognitive dissonance and the framing effect. Cognitive dissonance is when individuals reject information because of the psychological discomfort it may cause them if true – Tony may have rejected the idea that negative psychological experiences affect his CMP because this is commonly misunderstood to mean that the pain isn’t real [48] – but when reframed in the context of positive rather than negative psychological experiences, Tony’s beliefs change evidenced by his description of psychological euphoria with sports and reduced pain – there is no psychological discomfort caused by this belief. But this is purely speculative – it is beyond our scope to say exactly what cognitive biases led to these cognitive errors. However, it is worth noting that when the interviewer asked Tony about these contradictory beliefs, he admitted he hadn’t thought about it before, and subsequently questioned his own beliefs about psychological factors and pain: “They [negative psychological experiences] probably actually do [affect CMP], you know, negative do make a difference, yeah, thinking about it from that side of it… You probably don’t think that at the time, because, yeah, but yeah, yeah, I suppose it definitely could.”

### The mechanisms for formulating and justifying explicit beliefs about CMP

**Fig. 2:**
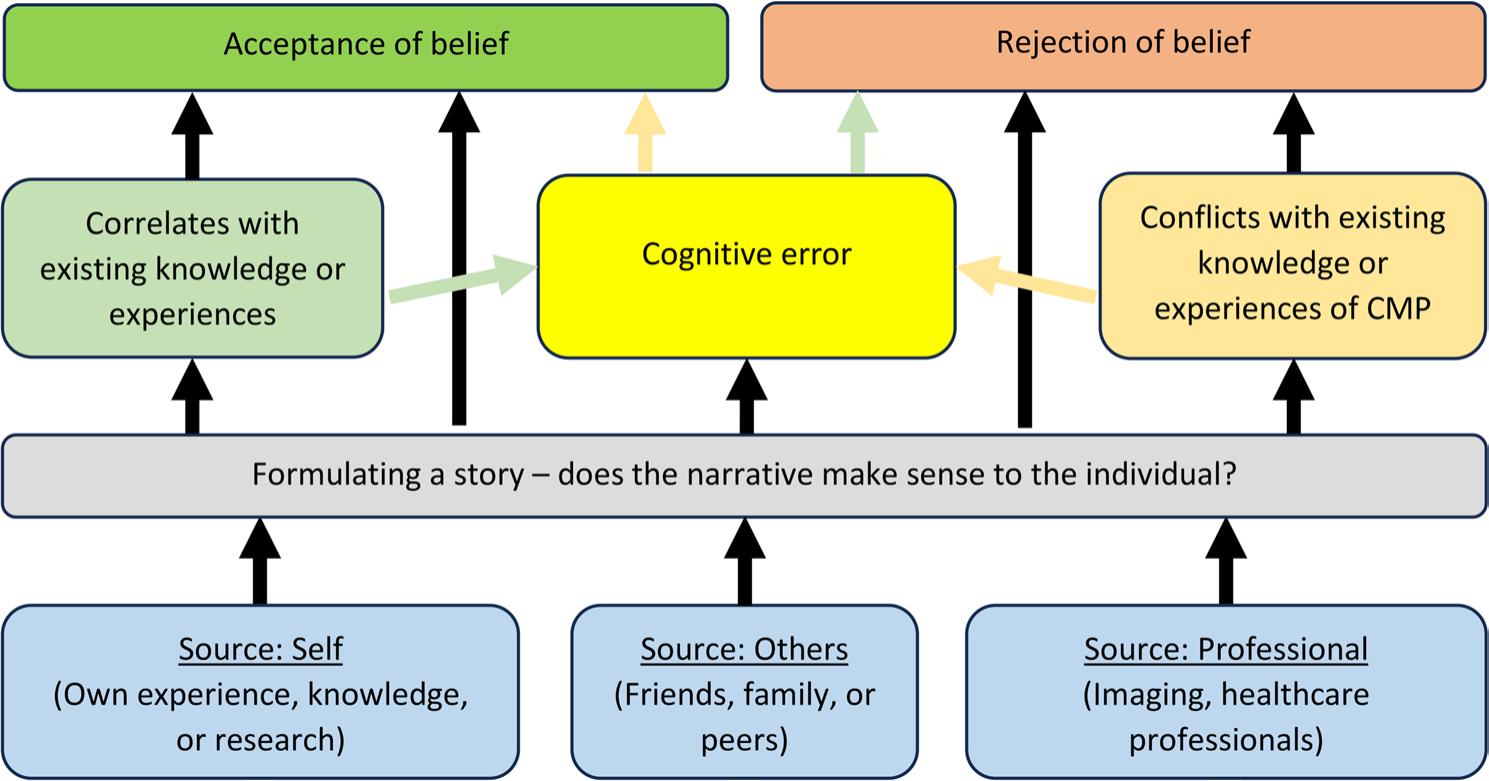
How explicit beliefs about CMP are formulated

Individuals’ explicit beliefs are grounded in multiple sources of information including themselves, others, or healthcare professionals with radiographic investigations. This information is synthesised by the individual into stories that they personally feel makes sense in understanding or explaining their pain, and this appears to be enough to accept beliefs, or reject narratives they feel do not make sense. When asked to justify their beliefs, individuals do so by checking against their existing knowledge or experience. To justify holding a belief, individuals provided examples where the story correlates with their experience (e.g., wearing heels must contribute to CMP because it hurts while wearing them) or existing knowledge (e.g., poor posture must contribute to CMP because of understandings of biomechanics and pressure). Likewise, to justify rejecting a belief, individuals provided examples of how the narrative conflicted with their experience (e.g., stress must not contribute to CMP because pain is not worse during high stress) or existing knowledge (e.g., psychological factors can’t contribute to CMP because they don’t cause structural damage). However, cognitive errors were apparent throughout this process meaning that some contradictory beliefs existed simultaneously, and that some narratives were rejected despite correlating with experience or knowledge (e.g., believing psychological factors do not contribute to CMP, despite pain being worse when psychological factors are worse). See Fig. 2 which outlines this process.

### How implicit beliefs about pain are formulated

We may assume that belief formation is purely an explicit, conscious process that happens in real time, in response to new information or experiences, that is grounded in logical reasoning. The reality is much more complex, with many mechanisms taking place beneath the surface of conscious awareness. For example, Murphy and Zajonc [49] demonstrated the power of affective priming by briefly displaying happy, angry or neutral faces, so quickly that they could not be consciously perceived, which significantly influenced subsequent evaluations of neutral stimuli (Chinese ideographs) that followed; participants liked or disliked the ideographs primed by the happy or angry faces flashed just before it. Not only do individuals form such beliefs without conscious awareness, but people also lack insight into these mechanisms, mistakenly assuming that their beliefs are the outcome of rational deliberation [45]. Once a belief is in place, however, individuals tend to construct retrospective justifications for holding it by detailing a logical narrative to explain the belief [50]. This happens even when the belief is implanted and in direct contradiction with recently stated beliefs [51]. These fallibilities in belief formation and justification are considered to be key reasons why individuals are susceptible to misinformation and cognitive errors [52]. These flawed mechanisms are thought to exist because the evolutionary function of reasoning is to devise arguments, not to evaluate the truth but to *persuade others*, as this is more important for thriving and surviving in social contexts [52].

### The mechanisms for formulating implicit beliefs about CMP

In our interviews, we were only able to observe the specific logical explanations individuals retrospectively used to explain and justify their beliefs, and the sources of information they cite for these justifications. We cannot directly observe the implicit, unconscious mechanisms of belief formation about pain because individuals are generally not aware of implicit beliefs and have little insight into how their explicit beliefs are formed [45]. Nonetheless, the authors are knowledgeable about the implicit processes of belief formation and therefore have interpreted this process within findings as part of our double hermeneutic analysis.

Pain can be amplified through implicit associations such as threatening language used to describe pain/injury [53], catastrophised interpretations of pain [6] or environments that reinforce danger or fear, that lead to unconscious, implicit beliefs that motivates certain behaviours and attitudes [54]. In our study, healthcare professionals were a salient source of explicit, justifiable beliefs about pain by providing diagnoses based on radiographic investigations, the foundations for an explicit belief. But these healthcare professionals imparted implicit beliefs too based on the language they used and how they framed their diagnosis. For example, Bethany recounts this conversation with a specialist: “and he says, so, but in 10 years-time you could end up in a wheelchair. I was like okay, so, I carried on, and then I stopped working in care, and I started to work in counselling. Because I was like, I need to change my career because I’m not going to be able to look after myself, never mind anybody else.” Explicitly, Bethany has been told she has degenerative disc disease, but implicitly she has received emotional cues that this is a highly threatening and dangerous disease, and as such develops beliefs that were not explicitly communicated, that she must avoid activity and change careers. Edward, on the other hand, recounts: “I did go to the GP about my knees, and she said ‘yeah, that’s osteoarthritis, nothing can be done about it, basically, just keep walking’ – I think, she said that.”. Explicitly, Edward has been told he has osteoarthritis, and implicitly he has received emotional cues of reassurance, that it is safe to remain active, and as such develops beliefs that activity is good for maintaining joint health. Furthermore, Edward, not being aware of the implicit origins of his beliefs, makes up a rational sounding justification for the belief retrospectively [44, 46]: “As these are living things, they presumably have the power to keep themselves repaired as much as possible… So, I believe that usage does continue to help the repair process, and non-usage tends to encourage it not to repair, and therefore to get worse. I just made that up, so I don’t know how clear that is.”

The above is an example of affective framing with emotionally valenced language, but there are numerous automatic processes that may contribute to implicit belief formation such as reinforcement learning, social modelling and other implicit motivations which create implicit beliefs about CMP that drive attitudes and behaviours, without conscious awareness [44, 46].

### The Dual Implicit-Explicit Processing (DIP) model of pain belief formation

**Fig. 3:**
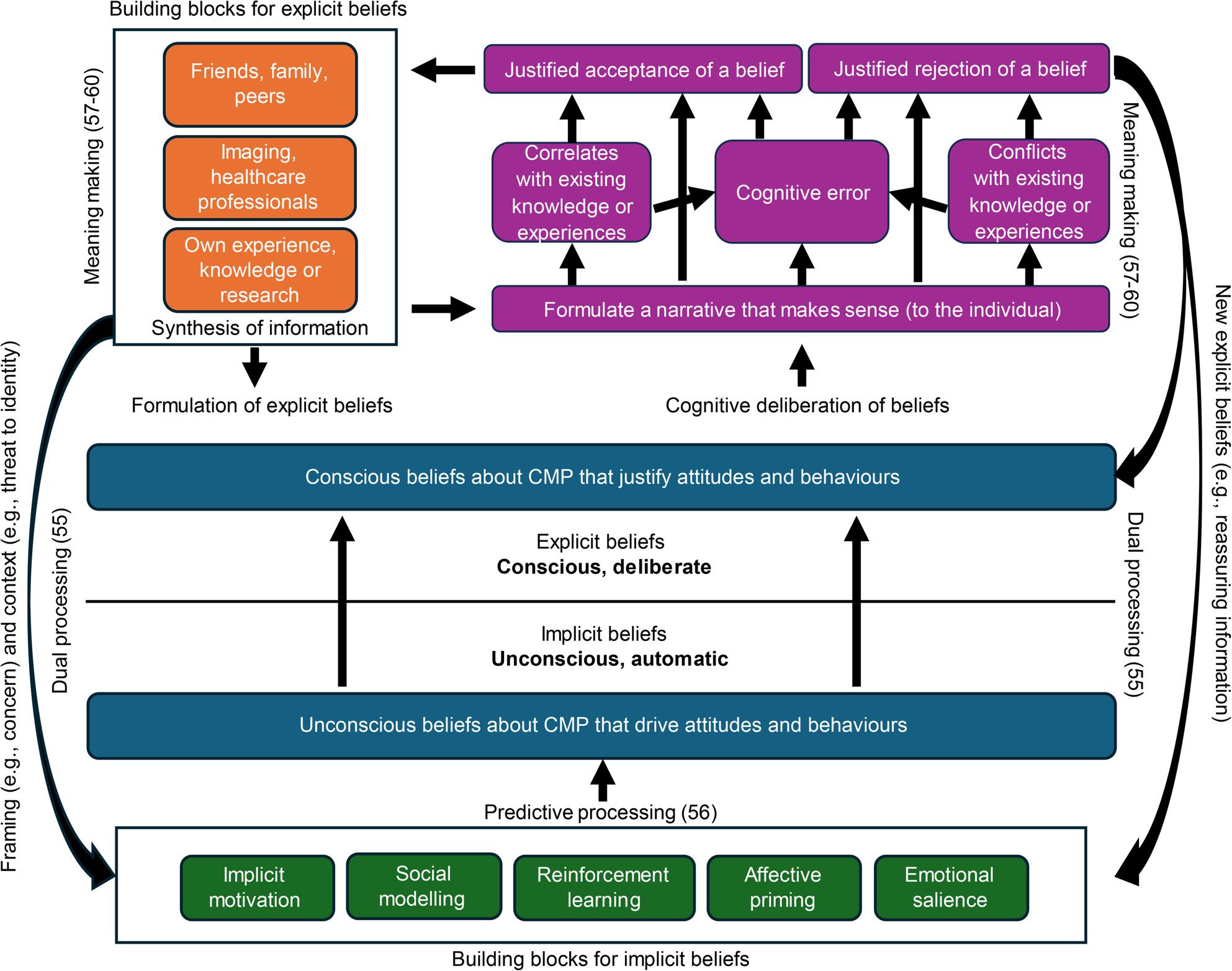
The Dual Implicit-Explicit Processing (DIP) model of pain belief formation

**Fig. 4:**
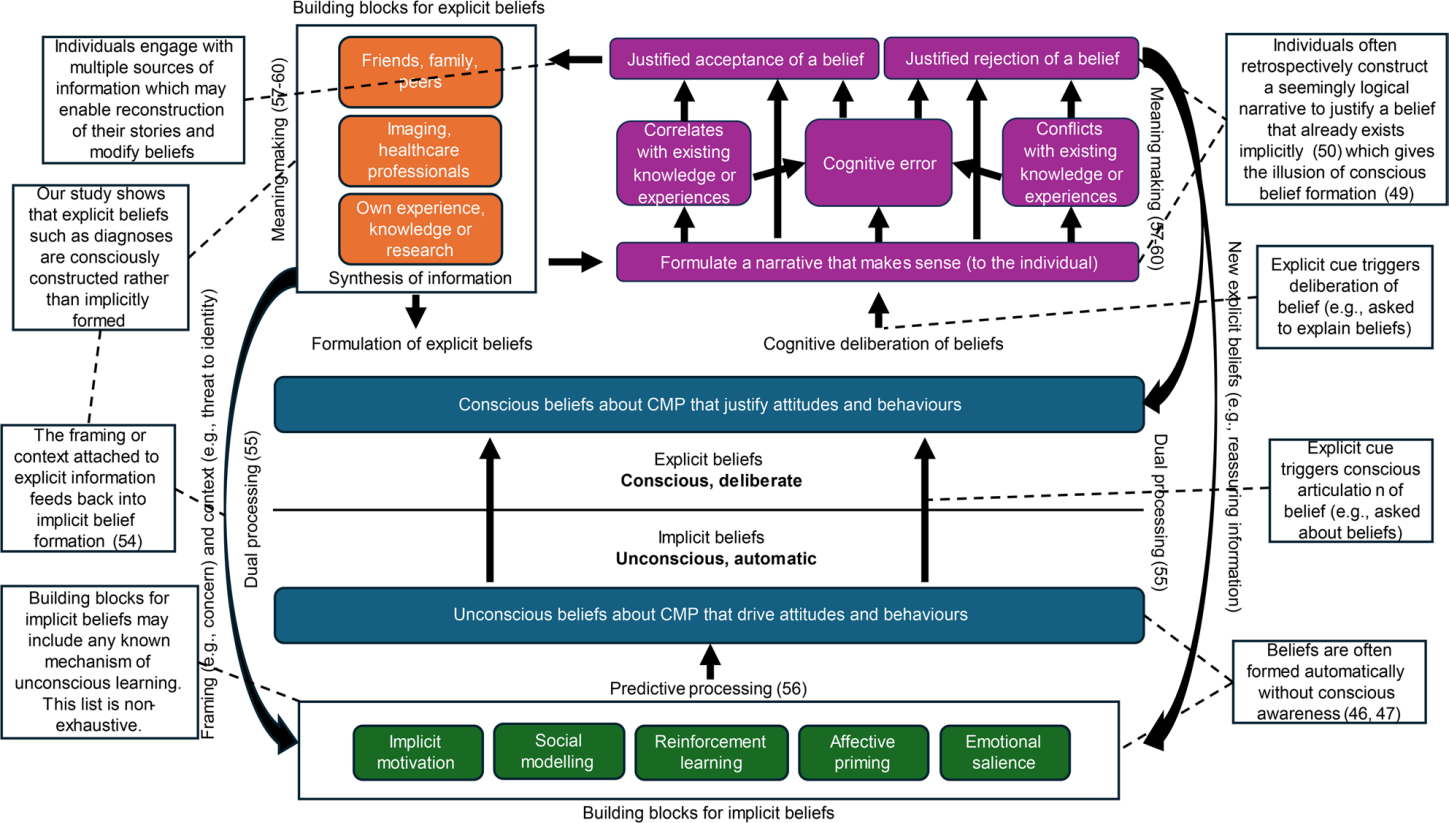
Annotated version of the DIP model of pain belief formation

The Dual Implicit-Explicit Processing (DIP) model of pain belief formation was designed to provide a visual representation of how individuals formulate their beliefs about CMP grounded in the findings from our study. The DIP model is a complete model of belief formation that integrates dual processing theory [55], predictive processing theory [56], and narrative constructivist meaning making theories [57–60] organised into implicit (automatic, unconscious) and explicit (cognitively effortful, consciously aware) processes. See Fig. 3 which outlines the DIP model. Fig. 4 is an annotated version of the DIP model which provides further explanations of mechanisms within the model.

## Discussion

This preliminary exploration study using IPA methods is the first study to explore how individuals formulate their beliefs on the biopsychosocial factors that contribute to their CMP. Participants use information from a number of sources in order to construct their own stories about their CMP; a key feature is that the stories must make sense to *them*. Participants justify beliefs by checking against their existing knowledge or experiences of having CMP, however cognitive errors were common, meaning participants held some contradictory or not logically justifiable beliefs (Fig. 2). Our interpretation of findings through the double hermeneutic includes a synthesis of directly observable explicit belief formation, with existing understandings of implicit belief formation, to provide a complete model of how people formulate beliefs about CMP: the dual implicit-explicit processing (DIP) model of pain belief formation (Fig. 3). These findings may serve as guidance for clinicians and researchers in helping individuals reconstruct implicit and explicitly held beliefs about pain, to better manage their CMP. Here, we will further situate the DIP model within this existing knowledge organised around cognitive processing – concepts around cognitive mechanisms – and meaning-making/self-understanding – concepts around the mechanisms of individuals’ interpretations of their own knowledge and experiences.

At the cognitive processing level, the DIP model aligns with dual-processing theories of cognition that differentiates fast, automatic processing which requires minimal controlled attention and working memory usage (referred to as type 1 processing), from slow, deliberate higher cognitive processing such as hypothetical thinking, mental simulation, and consequential decision making (referred to as type 2 processing) [55]. Drawing parallels with the DIP model, implicit beliefs about pain would be exhibited through fast, type 1 processing that guide perception and behaviour without awareness, whereas explicit beliefs arise when individuals recruit higher-order, type 2 processing to cognitively interpret their experience. This provides the foundation principles of the DIP model. However, integration with other models are required to consider the mechanisms that sit within these two types of processing to formulate beliefs about pain.

Predictive processing is a notable model that may account for the implicit learning aspect of the DIP model. Predictive processing is conceived as the brains effort to accurately interpret the world we live in through sensory input – primarily vision, hearing, touch, smell and taste, and actively synthesises this information to construct experience: our own individualised reality [56]. Based on this process, the brain holds internal models (or beliefs) for the best estimate of how the world works based on these senses and past experiences. When sensory data do not match predictions (e.g., pain with bending), the discrepancy prompts updating of working models of the world (or beliefs) (e.g., bending is harmful) – unless the brain assigns greater confidence to prior working models (e.g., established beliefs that bending is safe) [61]. This may be influenced by numerous other working models of reality such as current mental and physical wellbeing (am I currently well and able? ), possible impact of pain on life (will I be able to work, do things I enjoy, and maintain my important relationships? ), and beliefs about structural vulnerability of the spine (are the potential consequences of this severe? ). Much of this process is implicit – taking place without conscious awareness – and, depending on the perceived level of threat within predictive models, may automatically and unconsciously evoke feelings such as fear, behaviours such as avoidance, and a state of hypervigilance – CMP – rooted in an updated maladaptive model of reality characterised by the perception of threat within innocuous situations [61]. These implicit, unconscious, predictive models of reality are the antecedent of explicit beliefs, but how do these foundations develop into explicitly held beliefs?

We asked individuals what their beliefs were. This cue prompted individuals to actively and consciously articulate their implicit, working models of their individual reality, as explicit beliefs. Meaning-making and self-understanding literature may further explain the mechanisms individuals use for this aspect of the DIP model. Narrative constructivist theory posits that individuals organise experiences into narrative accounts that assign cause, coherence, and meaning [57]. We observed this within our study (theme 2 – stories that make sense). This can be expanded further into the concept of *narrative identity* – the internalised evolving story that integrates past, present, and anticipated future into a sense of self [59]. Within this framework, individuals confronting pain engage in explicit cognitive work to construct and revise narratives that explain their pain, justify its existence, and situate it within their broader sense of self. In our study, we observed this process to develop into adaptive beliefs for some individuals (self-reassurance, rationalising pain as not concerning, and perseverance of activity), and maladaptive beliefs for others (catastrophisation, avoidance, loss of self-identity). Further observed in our study, explicit belief formation is vulnerable to the impact of inaccurate or limited knowledge (theme 1) and cognitive error (theme 4). Hermeneutic phenomenology may further complement understandings of explicit belief formation. This posits that understandings are not simply acquired but *interpreted* through dialogue between the self and others (theme 1), and between new experiences and evolving predictions of meaning [58, 60]. This involves an interpretative negotiation: individuals test new explanations (from clinicians or social discourse) against their lived experience, accepting those that resonate with their bodily experiences and implicit understandings (theme 3), and rejecting those that do not (Fig. 2). In doing so, individuals seek coherence between what they feel implicitly, and what they believe explicitly, formulating a meaningful, coherent, communicable part of their life story.

In synthesis, the DIP model fits well within existing understandings of pain belief formation, and provides a unifying account that bridges dual processing, predictive processing, and narrative constructivist meaning making theories, offered as a full working model of how individuals formulate their beliefs about pain.

### Implications for practice

Pain education (i.e., targeted modification of explicitly held beliefs) alone is generally not effective in managing CMP [62, 63]. Instead, informed by the DIP model, we recommend targeting both implicit and explicit beliefs by integrating cognitive (explicit) education with experiential (implicit) learning to help patients align felt experience with new, more accurate models of pain.

#### Addressing implicit beliefs

Implicit beliefs may manifest in behavioural cues (e.g., guarded movement, avoidance) and subtle verbal cues (e.g., use of fearful or catastrophised language or excessively negative framing of pain), as opposed to explicit explanations. To address this:

- Use graded exposure, movement retraining, or experiential learning to gently challenge automatic responses and build new implicit models of safety and capability
- Use positive, encouraging verbal and body language to subtly encourage implicit reframing of pain
- Repetition and time are key, especially if maladaptive implicit beliefs are firmly established

#### Facilitating explicit meaning-making

Explicit beliefs will manifest through patients’ explanations of their stories and understandings. Clinicians should facilitate development of evidence-based knowledge by:

- Using reflective dialogue to help patients articulate and examine their beliefs by exploring where beliefs about pain originated (e.g., previous clinicians, social discourse) and how these shape current coping
- Encouraging narrative reconstruction by asking patients to tell their pain story, identifying turning points and reinterpreting them, to support adaptive evidence-based understandings
- Using meta-cognitive questioning to engage explicit (type 2) reasoning – have there been times where pain didn’t behave as expected? How does this fit within your pain story?
- Avoiding narrow, biomedical explanations based on radiographic investigations as these were salient, easy to remember, and used to justify maladaptive implicit models of pain

### Limitations and future directions

There are some limitations to this preliminary exploration IPA study. We sought to recruit individuals from a broad range of backgrounds which we were able to achieve in terms of socioeconomics, but notably it was difficult to recruit individuals from ethnic backgrounds with efforts to reach these individuals through social media unsuccessful. Research identifies a number of possible reasons for this including socioeconomic and logistical challenges and mistrust of healthcare/researchers [64]. In light of these issues, it is likely the research PPI mailing list we used for our main source of recruitment was under-representative of ethnic individuals. As such, future research should seek to recruit in settings with higher ethnic diversity, such as certain NHS services or community hubs, and consider the use of personalised recruitment approaches, whilst adhering to non-burdensome data collection methods as used in our study. Three of our six participants were in their 50s which may have affected results if identified beliefs were particular to this age group. We also did not recruit any individuals under the age of 40 with the most likely reason for this being that the prevalence of CMP increases with age with far fewer young people affected [65].

This preliminary exploration IPA study has identified a key avenue for future research. Particularly, future research should seek to better understand the relationship between implicit and explicit mechanisms for formulating beliefs about pain. Whilst we were able to outline the explicit beliefs held and conscious mechanisms used for their development, it was not possible to directly observe the implicit mechanisms of belief formulation within this study. We have therefore outlined the DIP model of pain belief formation in our discussion, based on our results combined with external research in the field of psychology. Further qualitative research is required to identify the specific implicit mechanisms used in formulating beliefs about pain that may fit within this model.

## Conclusion

To construct explicit beliefs about CMP, participants synthesised information from their own research, experiences and knowledge, and also conversations with friends, family and peers; but biomedical diagnoses orientated around structural damage or degeneration, evidenced by radiographic imaging, appeared to be particularly salient in formulating explicit beliefs about CMP. Participants wove this information into stories that explained their CMP. When why they held these beliefs, participants correlated the information with their experience or existing knowledge to justify accepting or rejecting beliefs; this process demonstrated instances of cognitive error. These findings are synthesised with existing knowledge on implicit belief formation to outline a complete model for formulating beliefs on CMP; the Dual Implicit-explicit Processing (DIP) Model of Pain Belief Formation.

## Supplementary Information

Supplementary Material 1.

Supplementary Material 2.

Supplementary Material 3.

Supplementary Material 4.

## Data Availability

All data generated and/or analysed during the current study are included in this published article and its supplementary information files, with the exception of the full interview transcripts which will be stored securely by University of Birmingham for 10 years, then destroyed in accordance with our ethical approval.

## Abbreviations

CMP: Chronic Musculoskeletal Pain
MSK: Musculoskeletal
COREQ: Consolidated Criteria for Reporting Qualitative Research
IPA: Interpretative Phenomenological Analysis
PPI: Patient and Public Involvement

## References

[1] Fayaz A, Croft P, Langford RM, Donaldson LJ, Jones GT. Prevalence of chronic pain in the UK: a systematic review and meta-analysis of population studies. BMJ Open. 2016;6(6):e010364. 27324708 10.1136/bmjopen-2015-010364 PMC4932255

[2] Vos T, Lim SS, Abbafati C, Abbas KM, Abbasi M, Abbasifard M, et al. Global burden of 369 diseases and injuries in 204 countries and territories, 1990–2019: a systematic analysis for the Global Burden of Disease Study 2019. Lancet. 2020;396(10258):1204–22. 10.1016/S0140-6736(20)30925-9 PMC7567026 33069326

[3] Foster NE, Anema JR, Cherkin D, Chou R, Cohen SP, Gross DP, et al. Prevention and treatment of low back pain: evidence, challenges, and promising directions. Lancet. 2018;391(10137):2368–83. 29573872 10.1016/S0140-6736(18)30489-6

[4] Bunzli S, Smith A, Schütze R, O’Sullivan P. Beliefs underlying pain-related fear and how they evolve: a qualitative investigation in people with chronic back pain and high pain-related fear. BMJ Open. 2015;5(10):e008847. 26482773 10.1136/bmjopen-2015-008847 PMC4611881

[5] DarlowB, Dean S, Perry M, Mathieson F, Baxter GD, Dowell A. Easy to Harm, Hard to Heal: Patient Views About the Back. Spine (Phila Pa 1976). 2015;40(11):842-50. 10.1097/brs.0000000000000901. 10.1097/BRS.0000000000000901 25811262

[6] Darlow B, Fullen BM, Dean S, Hurley DA, Baxter GD, Dowell A. The association between health care professional attitudes and beliefs and the attitudes and beliefs, clinical management, and outcomes of patients with low back pain: A systematic review. Eur J Pain. 2012;16(1):3–17. 21719329 10.1016/j.ejpain.2011.06.006

[7] Main CJ, Foster N, Buchbinder R. How important are back pain beliefs and expectations for satisfactory recovery from back pain? Best Pract Res Clin Rheumatol. 2010;24(2):205–17. 20227642 10.1016/j.berh.2009.12.012

[8] Arntz A, Claassens L. The meaning of pain influences its experienced intensity. Pain. 2004;109(1–2):20–5. 15082122 10.1016/j.pain.2003.12.030

[9] Zale EL, Lange KL, Fields SA, Ditre JW. The relation between pain-related fear and disability: a meta-analysis. J Pain. 2013;14(10):1019–30. 23850095 10.1016/j.jpain.2013.05.005 PMC3791167

[10] Alhowimel A, AlOtaibi M, Radford K, Coulson N. Psychosocial factors associated with change in pain and disability outcomes in chronic low back pain patients treated by physiotherapist: A systematic review. SAGE Open Med. 2018;6:2050312118757387. 29449945 10.1177/2050312118757387 PMC5808969

[11] Alhowimel AS, Alotaibi MA, Alenazi AM, Alqahtani BA, Alshehri MA, Alamam D, et al. Psychosocial Predictors of Pain and Disability Outcomes in People with Chronic Low Back Pain Treated Conservatively by Guideline-Based Intervention: A Systematic Review. J Multidiscip Healthc. 2021;14:3549–59. 35002245 10.2147/JMDH.S343494 PMC8722685

[12] Demmelmaier I, Åsenlöf P, Lindberg P, Denison E. Biopsychosocial Predictors of Pain, Disability, Health Care Consumption, and Sick Leave in First-Episode and Long-Term Back Pain: A Longitudinal Study in the General Population. Int J Behav Med. 2010;17(2):79–89. 19633960 10.1007/s12529-009-9055-3

[13] Vlaeyen JWS, Linton SJ. Fear-avoidance model of chronic musculoskeletal pain: 12 years on. Pain. 2012;153(6):1144–7. 22321917 10.1016/j.pain.2011.12.009

[14] Walsh DA, Radcliffe JC. Pain beliefs and perceived physical disability of patients with chronic low back pain. Pain. 2002;97(1–2):23–31. 12031776 10.1016/s0304-3959(01)00426-2

[15] NijsJ, Lahousse A, Kapreli E, Bilika P, Saraçoğlu İ, Malfliet A, Coppieters I, De Baets L, Leysen L, Roose E, Clark J, Voogt L, Huysmans E. Nociplastic Pain Criteria or Recognition of Central Sensitization? Pain Phenotyping in the Past, Present and Future. J Clin Med. 2021;10(15):3203. 10.3390/jcm10153203. 10.3390/jcm10153203 PMC8347369 34361986

[16] Caneiro JP, Bunzli S, O’Sullivan P. Beliefs about the body and pain: the critical role in musculoskeletal pain management. Braz J Phys Ther. 2021;25(1):17–29. 32616375 10.1016/j.bjpt.2020.06.003 PMC7817871

[17] LinIB, O'Sullivan PB, Coffin JA, Mak DB, Toussaint S, Straker LM. Disabling chronic low back pain as an iatrogenic disorder: a qualitative study in Aboriginal Australians. BMJ Open. 2013;3(4):e002654. 10.1136/bmjopen-2013-002654. 10.1136/bmjopen-2013-002654 PMC3641505 23575999

[18] Orhan C, Van Looveren E, Cagnie B, Mukhtar NB, Lenoir D, Meeus M. Are Pain Beliefs, Cognitions, and Behaviors Influenced by Race, Ethnicity, and Culture in Patients with Chronic Musculoskeletal Pain: A Systematic Review. Pain Physician. 2018;21(6):541–58. 30508984

[19] ReisFJJ, Nijs J, Parker R, Sharma S, Wideman TH. Culture and musculoskeletal pain: strategies, challenges, and future directions to develop culturally sensitive physical therapy care. Braz J Phys Ther. 2022;26(5):100442. 10.1016/j.bjpt.2022.100442. 10.1016/j.bjpt.2022.100442 PMC9550611 36209626

[20] Sharma S, Ferreira-Valente A, de Williams C, Abbott AC, Pais-Ribeiro JH, Jensen J. Group Differences Between Countries and Between Languages in Pain-Related Beliefs, Coping, and Catastrophizing in Chronic Pain: A Systematic Review. Pain Med. 2020;21(9):1847–62. 32044980 10.1093/pm/pnz373 PMC7553014

[21] Bada SO, Olusegun S. Constructivism learning theory: A paradigm for teaching and learning. J Res Method Educ. 2015;5(6):66–70.

[22] Hagger MS, Orbell S. The common sense model of illness self-regulation: a conceptual review and proposed extended model. Health Psychol Rev. 2022;16(3):347–77. 33461402 10.1080/17437199.2021.1878050

[23] Leventhal H, Phillips LA, Burns E. The Common-Sense Model of Self-Regulation (CSM): a dynamic framework for understanding illness self-management. J Behav Med. 2016;39(6):935–46. 27515801 10.1007/s10865-016-9782-2

[24] Joern L, Kongsted A, Thomassen L, Hartvigsen J, Ravn S. Pain cognitions and impact of low back pain after participation in a self-management program: a qualitative study. Chiropr Man Ther. 2022;30(1):8. 10.1186/s12998-022-00416-6 PMC8862196 35189908

[25] Walton DM, MacDermid JC, Giorgianni AA, Mascarenhas JC, West SC, Zammit CA. Risk Factors for Persistent Problems Following Acute Whiplash Injury: Update of a Systematic Review and Meta-analysis. J Orthop Sports Phys Therapy. 2013;43(2):31–43. 10.2519/jospt.2013.4507 23322093

[26] Dunn M, Rushton AB, Soundy A, Heneghan NR. Individuals’ beliefs about the biopsychosocial factors that contribute to their chronic musculoskeletal pain: protocol for a qualitative study in the UK. BMJ Open. 2022;12(7):e062970. 35863841 10.1136/bmjopen-2022-062970 PMC9310156

[27] Tong A, Sainsbury P, Craig J. Consolidated criteria for reporting qualitative research (COREQ): a 32-item checklist for interviews and focus groups. Int J Qual Health Care. 2007;19(6):349–57. 17872937 10.1093/intqhc/mzm042

[28] Stebbins RA. Exploratory Research in the Social Sciences (Vol. 48). Sage. 10.4135/9781412984249.

[29] Smith J, Osborn M. Chapter 4: interpretative phenomenological analysis. InQualitative methods in psychology: a research guide. Banister P, Burman E, Parker I, Taylor M, Tindall C, editors. London: Sage Publications; 2008. pp. 53–80.

[30] Brocki JM, Wearden AJ. A critical evaluation of the use of interpretative phenomenological analysis (IPA) in health psychology. Psychol Health. 2006;21(1):87–108.

[31] Guba EG, Lincoln YS. Competing paradigms in qualitative research. Handbook of qualitative research. Thousand Oaks, CA, US: Sage Publications, Inc; 1994. pp. 105–17.

[32] Schwandt TA. Constructivist, interpretivist approaches to human inquiry. Handbook of qualitative research. Thousand Oaks, CA, US: Sage Publications, Inc; 1994. pp. 118–37.

[33] Pietkiewicz I, Smith JA, editors. A Practical Guide to Using Interpretative Phenomenological Analysis in Qualitative Research Psychology. Psychological Journal. 2014;20:7-14.

[34] Smith JA. Reflecting on the development of interpretative phenomenological analysis and its contribution to qualitative research in psychology. Qualitative Res Psychol. 2004;1(1):39–54.

[35] Smith JA, Jarman M, Osborn M. Doing interpretative phenomenological analysis. Qualitative health psychology: Theor methods. 1999;1(1):218–40.

[36] Vasileiou K, Barnett J, Thorpe S, Young T. Characterising and justifying sample size sufficiency in interview-based studies: systematic analysis of qualitative health research over a 15-year period. BMC Med Res Methodol. 2018;18(1):148. 30463515 10.1186/s12874-018-0594-7 PMC6249736

[37] Smith B. Generalizability in qualitative research: misunderstandings, opportunities and recommendations for the sport and exercise sciences. Qualitative Res Sport Exerc Health. 2018;10(1):137–49.

[38] DunnM, Rushton AB, Mistry J, Soundy A, Heneghan NR. The biopsychosocial factors associated with development of chronic musculoskeletal pain. An umbrella review and meta-analysis of observational systematic reviews. PLoS One. 2024;19(4):e0294830. 10.1371/journal.pone.0294830. 10.1371/journal.pone.0294830 PMC10984407 38557647

[39] Braun V, Clarke V. To saturate or not to saturate? Questioning data saturation as a useful concept for thematic analysis and sample-size rationales. Qualitative Res Sport Exerc Health. 2021;13(2):201–16.

[40] Rodham K, Fox F, Doran N. Exploring analytical trustworthiness and the process of reaching consensus in interpretative phenomenological analysis: lost in transcription. Int J Soc Res Methodol. 2015;18(1):59–71.

[41] Kruglanski AW, Stroebe W. The Influence of Beliefs and Goals on Attitudes: Issues of Structure, Function, and Dynamics. In D. Albarracín, B. T. Johnson, & M. P. Zanna (Eds.), The handbook of attitudes (pp. 323–368). Lawrence Erlbaum Associates Publishers.

[42] Connors MH, Halligan PW. A cognitive account of belief: a tentative road map. Front Psychol. 2014;5:1588. 25741291 10.3389/fpsyg.2014.01588 PMC4327528

[43] Tullett AM, Prentice MS, Teper R, Nash KA, Inzlicht M, McGregor I. Neural and motivational mechanics of meaning and threat. In KD Markman, T Proulx, MJ Lindberg (Eds.), The psychology of meaning (pp. 401–419). American Psychological Association.

[44] GawronskiB, Bodenhausen GV. Associative and propositional processes in evaluation: An integrative review of implicit and explicit attitude change. American Psychological Association. 2006:692–731.Gawronski B, Bodenhausen GV. Associative and propositional processes in evaluation: An integrative review of implicit and explicit attitude change. Psychological Bulletin. 2006;132(5):692–731. https://psycnet.apa.org/doi/10.1037/0033-2909.132.5.692. 10.1037/0033-2909.132.5.692 16910748

[45] Nisbett RE, Wilson TD. Telling more than we can know: Verbal reports on mental processes. Psychol Rev. 1977;84(3):231–59.

[46] Olson MA, Fazio RH. Implicit attitude formation through classical conditioning. Psychol Sci. 2001;12(5):413–7. 11554676 10.1111/1467-9280.00376

[47] Savioni L, Triberti S. Cognitive Biases in Chronic Illness and Their Impact on Patients’ Commitment. Front Psychol. 2020;11:579455. 33192894 10.3389/fpsyg.2020.579455 PMC7655771

[48] Salomons TV, Harrison R, Hansen N, Stazicker J, Sorensen AG, Thomas P, et al. Is Pain All in your Mind? Examining the General Public’s Views of Pain. Rev Philos Psychol. 2022;13(3):683–98. 36164474 10.1007/s13164-021-00553-6 PMC9499913

[49] Murphy ST, Zajonc RB. Affect, cognition, and awareness: affective priming with optimal and suboptimal stimulus exposures. J Pers Soc Psychol. 1993;64(5):723–39. 8505704 10.1037//0022-3514.64.5.723

[50] Kunda Z. The case for motivated reasoning. Psychol Bull. 1990;108(3):480–98. 2270237 10.1037/0033-2909.108.3.480

[51] Johansson P, Hall L, Sikström S, Olsson A. Failure to detect mismatches between intention and outcome in a simple decision task. Science. 2005;310(5745):116–9. 16210542 10.1126/science.1111709

[52] Mercier H, Sperber D. Why do humans reason? Arguments for an argumentative theory. Behav Brain Sci. 2011;34(2):57–74. discussion – 111. 21447233 10.1017/S0140525X10000968

[53] Benedetti F, Lanotte M, Lopiano L, Colloca L. When words are painful: unraveling the mechanisms of the nocebo effect. Neuroscience. 2007;147(2):260–71. 17379417 10.1016/j.neuroscience.2007.02.020

[54] Wiech K, Tracey I. Pain, decisions, and actions: a motivational perspective. Front Neurosci. 2013;7:46. 23565073 10.3389/fnins.2013.00046 PMC3613600

[55] Evans JSBT, Stanovich KE. Dual-Process Theories of Higher Cognition:Advancing the Debate. Perspect Psychol Sci. 2013;8(3):223–41. 26172965 10.1177/1745691612460685

[56] Euler MJ. Intelligence and uncertainty: Implications of hierarchical predictive processing for the neuroscience of cognitive ability. Neurosci Biobehavioral Reviews. 2018;94:93–112. 10.1016/j.neubiorev.2018.08.013 30153441

[57] Bruner J. The Narrative Construction of Reality. Crit Inq. 1991;18(1):1–21.

[58] Gadamer H-G, Phenomenology, Hermeneutics M. J Br Soc Phenomenology. 1994;25(2):104–10.

[59] McAdams DP. Narrative identity. Handbook of identity theory and research, Vols 1 and 2. New York, NY, US: Springer Science + Business Media; 2011. pp. 99–115.

[60] Ricoeur P. Phenomenology Hermeneutics Noûs. 1975;9(1):85–102.

[61] Kiverstein J, Kirchhoff MD, Thacker M. An Embodied Predictive Processing Theory of Pain Experience. Rev Philos Psychol. 2022;13(4):973–98.

[62] Duhn PH, Wæhrens EE, Pedersen MB, Nielsen SM, Locht H, Bliddal H, et al. Effectiveness of patient education as a stand-alone intervention for patients with chronic widespread pain and fibromyalgia: a systematic review and meta-analysis of randomized trials. Scand J Rheumatol. 2023;52(6):654–63. 37162478 10.1080/03009742.2023.2192450

[63] Geneen LJ, Martin DJ, Adams N, Clarke C, Dunbar M, Jones D, et al. Effects of education to facilitate knowledge about chronic pain for adults: a systematic review with meta-analysis. Syst Rev. 2015;4:132. 26428467 10.1186/s13643-015-0120-5 PMC4591560

[64] Pardhan S, Sehmbi T, Wijewickrama R, Onumajuru H, Piyasena MP. Barriers and facilitators for engaging underrepresented ethnic minority populations in healthcare research: an umbrella review. Int J Equity Health. 2025;24(1):70. 40075407 10.1186/s12939-025-02431-4 PMC11905581

[65] Aili K, Campbell P, Michaleff ZA, Strauss VY, Jordan KP, Bremander A, et al. Long-term trajectories of chronic musculoskeletal pain: a 21-year prospective cohort latent class analysis. Pain. 2021;162(5):1511–20. 33230006 10.1097/j.pain.0000000000002137 PMC8054552

[66] World Medical Association Declaration. of Helsinki: ethical principles for medical research involving human subjects. JAMA. 2013;310(20):2191–4. 24141714 10.1001/jama.2013.281053

