# Supplementary material for: How individuals formulate their beliefs about chronic musculoskeletal pain: introducing the dual implicit-explicit processing (DIP) model of pain belief formation — a qualitative exploration
Source: BMC Musculoskelet Disord. 2026 Apr 29;27:518. doi: 10.1186/s12891-026-09835-5 (PMC13274004; doi:10.1186/s12891-026-09835-5)
Supplement: Supplementary file 3 — Supplementary Material 3. [file 12891_2026_9835_MOESM3_ESM.docx]

| **Stories that make sense to the individual** | Own experience, knowledge, or research | **Charlotte** [on origin of beliefs] “It comes from my experience, from my background. From my you know, my knowledge, you look up things”.  **Charlotte** [on origin of beliefs] “Oh, my knowledge of some science, and, you know, being brought up in a medical household, and just my understanding of the body… Well, I, I, did a I did a science degree. I did. I did a degree. I did physics, chemistry, and but I also did physiology, histology, and pharmacology. So, you know that's where my understanding comes from”.  **Tony** [on origin of beliefs around activity] “Experiences, yeah. Doing, becoming an athlete, as they call it, at my age, with all my conditions, you know. And then learning what's good and what's bad for you as a person, because everybody is different”.  **Tony** [on origin of beliefs about biological factors] “Being in the forces and being medically trained. So, I've got a bit of understanding from, because I was an R83, so I was, peels and clinical, so I've got, I think it's about, I just do a lot of research, you know. Google is a fantastic thing nowadays to find out research that's been done on a certain thing, and you know, you can get a better understanding now than before”.  **Bethany** [on origin of beliefs] “Just what I live through every day. And I did the research on degenerative discs when I first got diagnosed with it… and so I listen to my body quite a lot. So, it's listening to my body and just being aware of myself”. |
| --- | --- | --- |
|  | Healthcare professionals and imaging | **Bethany** “So when I saw the specialist, he did say to me, because you've left it so long… there's not a lot we can do apart from pain management… and then he says in 10 years-time you could end up in a wheelchair. I was like okay, so, I carried on, and then I stopped working in care, and I started to work in counselling. Because I was like I need to change my career because I'm not going to be able to look after myself, never mind anybody else”.  **Bethany** [on scans and diagnoses] “…and do a few more scans. Now it's gone up to my T7, T8, and in that time I got bursitis in my left hip. That’s when they found I got arthritis in that hip, and my knees, and my hands”.  **Bethany** “Yeah. So then I got diagnosed with the arthritis, and then on my last scan, I can’t remember when it was, a couple of years back, I was I was diagnosed with osteoarthritis in my back, in my spine, so obviously it's gotten worse”.  **Catherine** “I have had it X-rayed. So it's just arthritis”.  **Catherine** “in 2017 I had a big flare up. So I went to the doctors, and they sent me for an MRI, and that's when they discovered that I’ve got two degenerative disc disease in my L4 and L5”.  **Catherine** “I have a two x rays of my hip so, and I was horrified because the first one was like in my early 50s so I had a evidence [of osteoarthritis] when I gave up high impact stuff… And my recent X-ray was initially reported as normal. But the rheumatologist said well no, there’s joint space narrowing, but it's not got worse, so I'm hugely, you know, reassured by that”.  **Edward** “I did go to the GP about my knees, and she said ‘yeah, that's osteoarthritis, nothing can be done about it, basically’. ‘Just keep walking’ – I think, she said that”.  **Tony** “And now I got my MRI, was yeah, the L 2, 3, 4, 5, and S1 bulging”.  **Hannah** [when asked if “slipped discs” diagnosis came from healthcare professional] “Yes, yes, absolutely. Yeah. Saw a consultant, saw the scans, you know, had the options to whether to have surgery or not and… yeah”.  **Charlotte** “I think the scan that, well, that I think was an X-ray was done really to see about whether it was, whether I had, arthritis in my knees, which I have”. |
|  | Peers | **Charlotte** [on origin of beliefs] I have a brother who was a senior consultant, GP nephews and nieces”.  **Catherine** “what's happened to the rest of my family has also influenced me; because my sister is very overweight. She needed both her knees replaced at 50, but is so obese, is morbidly obese, so that absolutely has influenced my behaviour”.  **Hannah** “So yeah, some of those thoughts and beliefs are definitely from my childhood, either from my parents or from my schooling”.  **Tony** “the only reason why I went down a diagnosis route for that is because a friend… cause she had seen me at Invictus training camp, and she's saying to her husband ‘I think that Tony has got fibro’ etc., etc., you know. So then we had a good talk about it”.  **Bethany** “It helps talking to other people in the same boat with chronic illness, like on Facebook, living with chronic pain.” |
|  | Stories/narratives that make sense to the individual | **Charlotte** “the vertebrate were not even, and as it moved down. And, so you ended up with presumably cartilage on cartilage, but that presumably has worn away. And you've got bone rubbing on bone. That would be my interpretation of it”.  **Bethany** [on any other thoughts why she may have CMP] “my mum had me quite soon after my brother, and I think there's something to do with the fact that, they tell you not to have a babies for like a year after, and I was like a year and a month between me and my brother. I honestly think that there's something to do with that, the nutrients that obviously he stole when he was born. It wasn't resupplied before my mum got pregnant with me”.  **Bethany** “So my sciatica I know is down to my sciatic nerve that’s from my spine. So I understand that. The bursitis in my hip is when my bursa sac gets inflamed. And that can be because of the arthritis or because I’ve moved funny or overdone stuff”.  **Bethany** “I just think it's because of the degeneration. I think it's just getting worse. And then, obviously, as you get older all the things start to break down”.  **Hannah** [on why thinks “slipped discs” are cause of CMP] “They're pressing on something. And so yeah, I couldn't say. But having seen them, and seeing that they're bulging on to something that is triggering pain. Yeah, it must contribute to how I feel”.  **Edward** [on why CMP has continued] “Pain, is supposed to be a warning, isn't it? That something is not as it should be, and warnings are meant to tell you to change your behaviour, or to improve your behaviour, so presumably the damage that is there is not perfectly recovered from, therefore the warning signals still need to be sent out”.  **Edward** “I often wonder whether posture has anything to do with it. Poor posture encourages uneven load, for example, on knee joints. And poor gait, perhaps does the same”.  **Tony** [explaining what caused structures in spine to develop CMP] “Compression. I would say that was the most thing, compression, compression, compression. Because you’re always jumping, jumping, you know, always having loads on your back, always doing the very, very heavy work… So it's, yeah, there's a lot of heavy lifting and heavy, yeah, heavy compression work, so you know”. |
|  | Synthesis of information sources | **Edward** [on origin of beliefs] “I do have some knowledge of biology. And I do look things up on the Internet sometimes. Suppose the Internet wasn't around at the time that these things became my problems. But I suppose I could have read about it, probably talked about it, at least with the GP in the case of the knees, who may have said, and I've had time to say that the cause is, such and such. A lot is guesswork I think”.  **Charlotte** [on origin of beliefs] “Well, probably my medical knowledge, mainly. And seeing what other members of my family have, as well. So just it's clear from the family, you know, from my other family members, but also my medical knowledge. And X-rays, I’ve had X-rays”. |
| **Correlation with experience** | Easy to correlate with experience or knowledge (more likely to accept belief) | **Bethany** [on why believes degenerative disc disease is the cause of her CMP] “I just think it's the compression. I think it is down to the nerves, and the biology of it all. I don't think it's down, I don't think it's a psychological issue. I honestly do think it's down to, when I like tense up, so I can feel, just now I feel the pull” **MD** “so your experience marries up with the diagnosis of degeneration?” **Bethany** “Yeah”.  **Bethany** [elaborating on belief that she has poorer health because she was conceived too soon after older brother] “So I think he stole all my good genes, and the metabolism because he's skinny, he’s 42, he’s in my mum’s back garden now mowing, and he still plays football, and he has no issues”.  **Hannah** [on why she believes “slipped discs” are the cause of ongoing CMP] “Okay. So I believe that because whilst the pains got better, it's never gone away, and some of the pain I experienced in 2016, I still experience now. So that's what leads me to believe that there's a link between them, because it's been, some of it has been continual”.  **Hannah** [on why CMP continues] “partly because of the peri-menopause, the joy, the gift that keeps on giving. It all seems to have come together in the last couple of years. It started with the peri-menopausal symptoms, the fibromyalgia, and all of this”.  **Hannah** [on why believes stopping running and cycling was helpful] “I tried them and it hurt, and it didn't feel like a good hurt, you know, it wasn't comfortable, it wasn't pleasant. And there were other things I could do, so I'm not gonna do them”.  **Catherine** [on start of foot pain] “It’s to do with wearing heels I think because that's when I noticed it”.  **Catherine** [on why believes stopping activities was helpful] “I'd say purely because it was sorer when I did them”.  **Charlotte** [on why thinks walking helps CMP] “One time about 5 years ago, I think, I was really having problems. I don't know whether I pulled a muscle or done something, and I went [walking]. And yes, it did, that helped, a lot”.  **Edward** [when asked if thinks his approach to exercise has helped CMP] “It seems so. Yeah I’m certainly not disabled by it [CMP]”. |
|  | Difficult to correlate with experience or knowledge (more likely to reject belief) | **Tony** “I smoke, you know they will say ‘you gotta give up’ da da da, but is it a case of, my great grandad, he survived World War One, the Somme, etc., etc. He smoked the pipe till he was 96, drunk a whiskey in his tea every morning and he died at 96. So, it's hmm, okay. So it’s like, it is a lot to do with genetics”.  **Bethany** [asked if darker days (psychologically) contributed to CMP] “No, because I have less dark days now than I did before” [and CMP is similar].  **Catherine** [on why doesn’t think stress contributed to CMP] “I wouldn't say my pain was worse. So the time pain has been worse hasn't correlated with times of increased stress, I would say”. |
|  | Cognitive errors | **Catherine** [on reason believes stress doesn’t affect her CMP] “I'm not sure because that's not what I see in everyday life, you know, in my professional life [as a GP] I see that stress makes pain worse [for patients]. So I think my main belief is that I become more consumed with the stresses than the pain”.  **Catherine** [on cause of CMP] “My back I expect has become degenerative, I have got some changes on an x-ray as well, and, oh sorry and my hip, I’ve got osteoarthritis in my hip. So I think it's mainly degenerative… but obviously not in my thirties… I'm not sure… I'm not sure why my… well, no I still think it's just progressive and degenerative in nature”.  **Bethany** [asked if her psychological experiences contribute to her CMP] “No. I don't think so” **MD** “Is your pain worse on those [darker] days, or is it no different? **Bethany** “Probably worse”.  **Hannah** [on why carried on swimming but stopped running and cycling] “probably something I’ve never admitted out loud, swimming does aggravate my neck sometimes… I've carried on swimming, regardless of the fact that it can and does aggravate [symptoms]. **MD** “and you’ve done that because you want to keep swimming?” **Hannah** “Yes, because I love swimming”.  **Tony** [explaining that negative psychological experiences do not affect CMP] “I don't, I don't think they knock on as much. They probably do… affect the mental side more than the physical. Definitely, yes, but yeah, not the other way around”. [later explaining that positive psychological experiences do affect CMP] “Yeah, because when you’re focussed on something and you're feeling, you’ve got them endorphins going around, you know, pain goes away, which is very strange. The pain, you know it’s still there, but you can't feel it. You're not, because you're not concentrated on that pain, you’re concentrating on the good things, the things that's making you happy. The pain goes away”. |
